# I am no longer alone – How do university students perceive the possibilities of social media?

**DOI:** 10.1080/02673843.2014.919600

**Published:** 2014-06-05

**Authors:** Satu Uusiautti, Kaarina Määttä

**Affiliations:** ^a^ Faculty of Education, University of Lapland, Rovaniemi, Finland

**Keywords:** social capital, social media, social networking site, university student, social agency, peer support

## Abstract

An increasing number of people have become users of social media, mostly looking for social contacts and networking. But what kind of social capital do social networking services (SNSs) provide? University students' (*N* = 90) experiences of and opinions on social media were studied through a semi-structured questionnaire. The following research questions were set for this study: (1) What kinds of benefits do university students perceive in the usage of social media? and (2) What kind of social capital does social media produce according to university students' opinions? Their answers were analysed with the qualitative content analysis method. The results revealed that SNSs can increase students' social capital in many ways, such as in the form of peer support groups and learning environments, and enhance bonding and communality in them. These possibilities should be better studied in educational contexts, as they can have a positive impact on students' well-being, engagement to studies and, thus, study success.

## Introduction

Humans are social by nature. People spend an increasingly considerable time on social media or social networking services (SNSs); writing about one's doings, sharing photos and following others have become a salient part of our lives (boyd & Ellison, 2007). In this paper, we are, on one hand, interested in the benefits of social media in general and, on the other hand, in the educative uses of social media specifically. Selwyn (2009, p. 158) has noted that ‘the prominence of SNSs in the lives of learners of all ages has prompted great enthusiasm amongst some educators’. He refers to educators' willingness to take advantage of this pertinent part of children's and youth's lives in teaching and education. According to Lindstrom (2012), the effort of integrating technology in teaching and classroom activities has, however, remained rather superficial thus far. In order to ignite meaningful engagement with subjects in students via SNSs, teaching and education need cultural changes (Carey, 2013). On the other hand, some studies have suggested that the use of SNSs in education support student involvement with the subject and to develop basic skills and contribute to a higher engagement with the subject and a deeper collaboration with other students and teaching staff (Arquero & Romero-Frías, 2013; Mason, 2006). Tian, Yu, Vogel, and Kwok (2011) suggest that students' online social networking is straightforwardly influential to their social learning, while the influence on the academic learning should be further studied.

In this study, we were interested in analysing what kinds of opportunities or benefits university students in a Finnish university consider for SNSs, especially education-wise. However, the purpose is not so much to discover new pedagogical solutions, but to increase understanding of the nature of using social media and its possible influence on students' lives, and in this way support their success in studies (see for example Määttä & Uusiautti, 2011; Uusiautti & Määttä, 2013).

Indeed, international research on people's behaviour in social media has exploded and provided numerous points of views that are sometimes even contradictory (see for example boyd & Ellison, 2007; Caers et al., 2013; Haythornthwaite, 2005; Rohn, 2014). What is certain, however, is that SNSs have influenced and will influence human beings' social lives. Some studies suggest that people who actively participate in Facebook are more likely to experience connectedness and feel happier (Valkenburg, Peter, & Schouten, 2006). Students with lower levels of life satisfaction tend to seek to participate in online networks to increase their personal well-being (Ellison, Steinfield, & Lampe, 2007).

Steinfield, Ellison, and Lampe's (2008) study combines the idea of social capital with human well-being and SNSs. Their viewpoint rests on the idea that social capital is illustrated in social relationships as presented by Bourdieu. Bourdieu (1996) divided capital into three types: cultural capital, economic capital and social capital. The first refers to an individual's concrete, financial capital, possessions and ownership (money, things, income and wealth), while cultural capital means the ownership of cultural products, a certain way of life and making choices as well as the ability to make use of and produce culture (including education). Social capital is the entity of those actual and potential resources that are connected to social relationships and the ability to mobilise people (Bourdieu, 1984). Social capital is immaterial and connected to mutual recognition and appreciation among individuals.

How is social capital connected to social media? Notwithstanding, social ties make a key feature of individuals' life satisfaction (Kahneman & Krueger, 2006), and in today's world, social media has become an important channel of increasing social capital (Ellison et al., 2007). Kavanaugh, Reese, Carroll, and Rosson (2005) distinguish two types of social individuals in SNSs: bridging individuals, who create and organise networks, and bonding individuals, who establish close relationships with others, for example, within a network. The researchers' findings suggest that bridging individuals can enhance social relations and information exchange, and increase face-to-face interaction via SNSs. All this helps to build both bonding and bridging social capital in communities (Kavanaugh et al., 2005). Bridging social capital refers to loose connections between individuals who may provide useful information or new perspectives for one another, but typically not emotional support like bonding networks do. Likewise, Ellison et al. (2007) discovered that the use of social media has a strong association with social capital and interacts with measures of psychological well-being in college students.

In all, SNSs, thus, make an important channel of establishing and maintaining social relationships and have become an important element of human well-being and happiness as well (Saslow, Muise, Impett, & Dubin, 2012; Seder & Oishi, 2012). Here, it is important to refer to the fourth capital type developed by Luthans, Luthans, and Luthans (2004). The concept of positive psychological capital adds to the discussion the element of psychological well-being (see also Uusiautti & Määttä, 2014b) and is closely connected to the human being's social nature (e.g. Berscheid, 2006). These researchers claim that knowing ‘who I am’ is as equally important as ‘what I know’ and ‘who I know’ (Luthans et al., 2004). However, it seems that in the world of SNSs where social relationships are created and maintained through our self-built identities (Seder & Oishi, 2012; Valkenburg et al., 2006), these capitals are closely interconnected.

## Method

This study attempts to analyse what kind of benefits university students see for social media, how they use them, and how they think they could be used. These themes were studied through the following research questions:

1. What kinds of benefits do university students perceive in the usage of social media?
2. What kind of social capital does social media produce according to university students' opinions?

The study was conducted on 23–25 September 2013 among students at a northern Finnish university participating in an educational psychology course entitled ‘The Basics of Learning and Developmental Psychology’ (5 ECTS). The course lecturer asked the participants to fill out a questionnaire about their relationship with social media. Answering was voluntary and anonymous, and so did not influence, for example, the students' grading.

Of the 140 students taking the course, 90 students were participated in the research by returning the questionnaire, a relatively good participation rate of 64%. Only seven respondents were men (7.8%) and 83 were women (92.2%). This uneven gender balance lies in the fact that students of education and educational psychology are mostly women. However, because the purpose of the study was not to discover any gender-specific differences in the use of social media but only students' SNS behaviour in general, the data were considered suitable, and would not be analysed in terms of gender.

The questionnaire included both structured questions and open-ended questions. The structured questions were for collecting background information only, while the answers provided for the open-ended questions formed the actual data. The answers were analysed through qualitative content analysis in the light of the research questions (see for example Mayring, 2000). The questions focused on four core areas: (1) the students' opinions on the social media and its importance, (2) their behaviour in the social media, (3) rules of using the social media and agreements between friends and family members and (4) the usability of the social media. This article discusses university students' answers in themes 2 and 4 for the educational utilisation of SNSs.

Qualitative content analysis method was utilised in this study. According to Mayring (2000), categories are, thus, in the centre of analysis. The research questions direct analysis and aspects of text interpretation are put into categories. In this study, the themes that emerged from the data were used as the categories and sub-categories forming the results. According to the procedures of content analysis, the categories were carefully founded and revised within the process of analysis; this is called feedback loops (Mayring, 2000). This analysis method was considered suitable because the main interest was in the contents, the ways the participants described themselves as users of social media and their practice-based perceptions of its benefits and usability, rather than in numerical information (see also Creswell, 2007). However, some information about the amount of participants illustrating each content category are given to show that the interpretations are not based on just singular answers in the data.

The background questions covered the participants' age, gender and marital status. They were also asked whether or not they used the social media and, if yes, how often. The participants were then asked to describe briefly their most important reason for using social media. Table 1 illustrates the participants' background information.

**Table 1:** Participants of the SNS survey.

Category	Specification	*N* (%)
Gender	Women	83 (92.2%)
	Men	7 (7.8%)
Age	Under 20	22 (24.4%)
	21–30	48 (53.3%)
	31–40	16 (17.8%)
	41 and over	4 (4.4%)
	Mean age	25.8
Marital status	Single	35 (38.9%)
	Dating	23 (25.6%)
	Married/co-habiting	32 (35.6%)
Uses social media	Not at all	2 (2.2%)
	Once a month	0 (.0%)
	Once a week	2 (2.2%)
	Several times a week	4 (4.4%)
	Once a day	10 (11.1%)
	Several times a day	72 (80.0%)

The data obtained in this study were relatively biased due to the student group recruited; a vast majority of the participants were women and even quite young ones. When it comes to the reliability of the study, the data were considered suitable because almost everyone used SNSs and were, at least, familiar with them. The answers given in the questionnaire did not illustrate just the participants' ideas but they were also based on their practical experiences in SNSs. Relatively many of the participants described their relationship with social media as natural; a SNS or SNSs had become a part of their everyday lives and the classification between ‘the real life’ and ‘SNS life’ had become vague. Thus, the data provided a good way of analysing the benefits of social media according to these university students' points of view.

## Results

### Utilisation of the social media for among university students

University students were asked to describe their personal usage of the social media. The purpose was to find out how widely the students use SNSs in their lives and if they had specific educational reasons for the use. As Table 1 showed, nearly all students participating in this study were familiar with the social media. The majority of them (*N* = 84) reported that they used the social media for social purposes; for maintaining social relationships and communication with friends, relatives, family members and other social. Other important reasons for using the social media were information seeking, sharing, and distribution (*N* = 19). The rest emphasised personal amusement and pastime.

When considering the social use of SNSs, a majority of students reasoned the importance with the easiness and speed of interacting and communicating with others. SNSs provided them with new ways of creating and maintaining networks with various purposes.

If it leads to respectful, honest, and awareness-widening discussion, and its influence is good…. (Student No. 34)

SNSs is especially important for making social interaction easier and constructing communality. (Student No. 33)

In all, the students' evaluations about their reasons for using the social media were mostly connected with social relationships and interaction. Most of the students justified the use of the social media with the increased and easy interaction with friends and relatives even around the world (see also Rohn, 2014). This also meant that they had taken the social media as a part of their real life, their everyday doings and daily chores.

I visit the social media many times a day, and it is a really good way of keeping in touch with people and follow others' doings and happenings. It is a crucial part of my day nowadays. (Student No. 30)

However, the students did not forget the dangers of the social media either. We have discussed these in detail in our earlier study (see Uusiautti & Määttä, 2014a). For the point of view of this study, it is noteworthy that students were concerned of the time that the usage takes from ‘the real life’, the actual face-to-face communication with people. They were also worried about the influence on people's identities and possible occurrence of double identities: the real-life and online identities. Many students called for increasing information about the social media and, for example, education about the usage and both benefits and risks related to SNSs.

I check that pictures and texts are appropriate and give a good ‘impression’ of me… I usually think about texts carefully; I want them to bespeak of me and no one else. (Student No. 36)

They function as identity-builders to many people very powerfully. Some people go to extremes and they create an ideal identity in the social media, and no one can recognize the same in these persons in live world. (Student No. 35)

### Benefits of the social media according to university students' opinions

When asking university students how they perceive the opportunities of employing the social media more efficiently, their viewpoints varied from ‘no more possibilities/the social media has reached it top usage’ (*N* = 10, 11.1%) to detailed descriptions of various utilisation possibilities (*N* = 51, 56.7%). Others chose not to answer this question.

Those who were able to see further opportunities for the usage of SNSs could be categorised roughly into four categories based on the themes emerging from their answers.

#### In education

Most ideas (*N* = 22) concerned the use of social media in education. Students were certain that the opportunities of using the social media in education are ‘limitless’ (Student No. 88). They mentioned study groups that could enhance cooperative learning; either students or teachers could form groups around a theme or a school subject in which students could share and discuss the study theme, develop projects or ask questions and give answers. Teachers could post assignments and give directions via SNSs, and students would have access to these tasks from anywhere and anytime. Other study-related information, such as scheduled exam dates or information about excursions, could be posted in the social media networks.

The social media can be well employed, for example, in education. Students created environments for themselves in the social media, and share information and their experiences there. (Student No. 77)

It could be probably used in teaching. Courses could have close communities that would enable students' interaction also out of school hours. (Student No. 7)

Indeed, social media was considered an important future channel of interaction at school, and some of the students mentioned that it would be necessary to not only use SNSs at school for better and more efficient interaction, but also to teach modern interaction, the interactive side of it, which has become increasingly important in today's life to everyone.

Opportunities to teach people to interact virtually, and it would develop people's writing and influencing skills as well. (Student No. 57)

It could be used more at work and school, because it offers good possibilities for being in contact with people. (Student No. 73)

Furthermore, interaction via SNSs in education would not be limited to just students and teachers but would include also parents and partners outside school, such as local entrepreneurs. The social media would, thus, enhance knowledge of the local and global environment and society; SNSs make learning environments of their own.

The social media could be used more in the home-school cooperation. (Student No. 77)

It makes global interaction possible at school. (Student No. 17)

The social media can make use of other people's knowledge. You can learn via SNSs. (Student No. 5)

#### For bonding and peer support

The second most important future usage of the social media mentioned by university students was its possibilities for enhancing bonding and providing communal support (*N* = 15). Students emphasised the opportunities of bonding as well as building and learning communality in social media. This advantage was reasoned with the easiness of contacting and interacting with others via SNSs and, thus, the incomparable ease of creating networks.

For stirring experiences of bonding! (Student No. 57)

People could use the social media more rationally especially for networking, and, thus, considering their future, too. (Student No. 82)

An especially invaluable feature of SNSs was, according to students' opinions, peer support groups that could be used better and more widely in the future.

It is particularly good for peer support groups that kind of offers anonymity but still you can talk and share, and give and receive support from people experiencing the same. (Student No. 72)

It can provide a support network, e.g., for weigh management, new acquaintances, group activities, peer support, online second-hand shops, village communities, etc. (Student No. 54)

In all, bonding and communality, as well as group activities, in the social media were considered important ways of finding positive experiences. In today's world, people spend plenty of time on SNSs, and therefore, the human ingredient, sense of togetherness and happy experiences provided via the social media, are becoming more and more important for human well-being. However, it is not self-evident that everyone can benefit from the social media like this. Therefore, education can be crucial:

In my opinion, an individual people has the opportunity to get positive experiences from the social media, if the individual knows how to realize the opportunities. (Student No. 71)

#### For awareness and information sharing

Some of the students (*N* = 9) considered that the social media could be used more effectively for information sharing and increasing awareness about social issues and other topical events and matters. They emphasised that plenty of important information can be missed without SNSs where one can learn about something that is not reported by the traditional news media. In this way, the social media also could function for giving voice for those treated unfairly, and therefore, for good-doing.

The social media could be used more for bringing out issues needing a change. It is a place where you can reach plenty of different people from different places, which makes it possibilities for good – quite significant. (Student No. 45)

Informing about good things and soft values and reaching masses in cases of emergency, making influencing on social matters easier, and letting the voices of people facing inequity heard. (Student No. 49)

#### For worklife

Rest of the students' answers (*N* = 7) were focused on worklife usage of social media. They referred mostly to marketing and communication opportunities, but also had noticed the possibilities for ideas sharing and cooperation and for finding a workplace. Enterprises not appearing in SNSs were considered outdated, while certain professionally oriented social networks were seen important in finding employment and creating work-related networks.

The possibilities of marketing and communicating are limitless – not many enterprises or communities know how to make use of it! Social media is, however, an easy way of reaching a big audience; when used in a right way, it makes a powerful means. (Student No. 48)

## Discussion

When summing up university students' experiences and opinions on the utilisation of social media, one factor becomes the most essential: its social benefits. This is in line with the findings about two fundamental reasons for joining social media: (1) the need to belong and (2) the need for self-presentation (Nadkarni & Hofmann, 2012). Likewise, Lee, Yen, and Hsiao (2014) showed that, among university students, experiential value is found to be most significant in social media; users seemed to mostly fulfil their psychological needs, such as sharing the useful information and receiving enthusiastic replies or praise, from their social contacts on SNSs.

In this study, the students described that their main action on SNSs is focused on social relationships and friendships and that the networks existing in social media are one of the main reasons for using social media. Notwithstanding, the mentioned threats and dangers of social media were also connected with social relationships and the possible damage it can do to them.

Then, we wanted to know how the university students thought social media could be used better or more efficiently. In these answers too, their emphasis was on communality, bonding, group activities and peer support; they all can be seen as components of social capital. While some students talked about various methods of using social media, for example, as a part of education or to create special focus groups, some others referred to possibilities of enhancing awareness and sharing experiences and information. All these possibilities were connected to the need and wish to work for everyone's good, to improve social interaction, and to provide positive communal experiences to each other.

What can be then said about the social capital constructed in social media? What is it like? As the results showed, students considered social media as a way of enhancing the positive sense of togetherness and constructing communality that are based on the internet. The sense of giving and receiving support, thus, differs from what one might have in real-time face-to-face encounters, but is not any less important. Especially, when thinking about the increasing demands on individualism and self-directed action necessitated from today's people by schools, work life and other areas of life, it is all the more important the discuss the social opportunities and benefits of the modern life styles. Furthermore, as the students highlighted, bonding does not mean only support of special interest groups but information sharing in general and wide educational and work life connections. They also mentioned influencing and active civic participation (cf., Valenzuela, Park, & Kee, 2009). Likewise, the multidimensional educative nature expands from widening classroom contexts to home–school collaboration. Next, we will discuss these viewpoints in detail.

## Conclusion

First of all, the findings of this study are interesting if we think that one of the major concerns related to social media cover narrowing human interaction and perceived loneliness (Kraut et al., 1998; Odaci & Kalkan, 2010; Shapira, Goldsmith, Keck, Khosla, & McElroy, 2000). On the other hand, there are increasing studies showing the benefits of peer support networks that make communicating with others easier and that are encouraging further research and applications for such usage (Oldmeadow, Quinn, & Kowert, 2013; Przybylsk & Weinstein, 2012). It has been proven that interactions with peers who are successfully coping with their problems are more likely to result in positive behaviour change in others (Solomon, 2004). In Takahashi et al.'s (2009) study, participants felt empowered by giving online peer support to others in SNSs. The findings are supported by various studies researching specific phenomena, such as one by Vaarala, Uusiautti, and Määttä (2013) reporting that online peer support can be very important for college students' coping with loneliness.

The role of internet and online communication has been known already a while in adolescents' lives (e.g. Scherer, 1997). In schooling contexts, this area of life must be acknowledged and taken seriously (Silius et al., 2010). Social media provides many opportunities for education: it can function as a learning environment, a student peer network and a medium of learning new, relevant and topical skills. SNSs can be, thus, used for education and in education more efficiently.

First, social media can be considered a new type of a learning environment providing both formal and informal learning and education opportunities (e.g. Niemi & Multisilta, 2014). Not only did university students perceive social media as a suitable and functional place for discussing themes studied at school with peer students and teachers, but also with others. According to Mason (2006), this type of possibility to timely and context-bound feedback in education has potential to deliver a positive learning experience (see also Selwyn, 2009). Furthermore, one of the crucial problems in university-level studies is that the beginning of studies is the most difficult for many students also because they do not yet know their peers or teachers (Määttä & Uusiautti, 2011). SNSs could be used for lowering the doorstep for contacting and familiarising them.

Furthermore, SNSs could be used for learning from other relevant people or communities about the theme at hand, for example, by inviting worklife representatives to tell about their experiences and viewpoints and including an opportunity to ask questions from them. When used as a learning environment, tasks, schedules and other relevant information about studies could be shared through SNSs.

Some pedagogical applications are already introduced, such as a personal learning environment, which is a potentially promising pedagogical approach for both integrating formal and informal learning using social media (Dabbagh & Kitsantas, 2012). The study by Dabbagh and Kitsantas (2012) showed that learning in social media support students' self-regulated learning in higher education contexts (see also Mason, 2006; Valtonen et al., 2011).

A particular application of SNSs is student networks. These close communities can be easily established and they enable students discuss about specific courses, themes, student life, or whatever topics the communities were based on or built for. This kind of sharing can be seen not only enhancing students' study success but also their engagement in studies in addition to their mutual social networking and relationships (e.g. Steinfield et al., 2008). At its best, students can learn how to act as self-organising, proactive, self-reflecting and self-regulating individuals within a broad network of socio-structural influences (see also Bandura, 2001a, 2001b).

The third viewpoint to the educative use of social media relates to various skills that are required in today's world, often referred as literacies. There are increasing studies about the benefits of using social media not only to enhance learning of the school subject, but also to develop interaction skills when it comes to expressing oneself by writing in social media. For example, Chen (2012) studied how students of second language (L2) learned social network communication skills in internet-mediated social and communicative contexts. These skills are important for today's students and make an important part of their SNS-based social capital too. Without ability to critically reflect on and efficiently express oneself in social media, SNSs are neither likely to benefit the user nor increase his or her social capital.

When it comes to education, the important implication from this study was that teachers could act as bridging individuals creating possibilities for bonding among students and, thus, enhance the sense of meaningful studies and have a positive impact (e.g. Cruce, Wolniak, Seifert, & Pascarella, 2006; Uusiautti & Määttä, 2013). As the role of a teacher as a mentor has become more and more important and versatile in these days, SNSs can provide a useful way for the teachers' bridging role. According to one study (Kunttu & Huttunen, 2008), only a little over half of university students feel like belonging to some study group. About a third of them do not feel like belonging to any group. In Lähteenoja's (2010) research, over half of new university students had never discussed scientific questions, or their studies, difficulties or future plans with teachers. Indeed, Kezar and Kinzie (2006) emphasise that focusing on the early years of study and respect for various learning styles and methods are keys to successful and meaningful study paths (see also Kuh, Cruce, Shoup, Kinzie, & Gonyes, 2008). Here, we see the possibilities of SNSs in education as the place for encounter, information sharing and distribution, and as a way of creating social relationships and increasing students' social capital.

When viewing the possibilities of SNSs in education at basic education level, yet another interesting opportunity lies in its usability for home–school cooperation. This was mentioned by university students in this study, but the idea certainly deserves to be more closely studied (see also Uusiautti, Määttä, & Määttä, 2013). According to Hoover-Dempsey and Sandler (1997), parental involvement in education has long been a topic of interest among those concerned with optimal developmental and educational outcomes for student. Such practices mean that both school and home pursue developing self-esteem and health in students (e.g. Desjardins, Zelenti, & Coplan, 2008). Perhaps, SNSs in education could provide an accessible means for parents to get involved in their children's education and communicate with teachers, too.

Behaviour in the social media, as in the everyday life in general, involves a high level of internalised reflection (Putman, 1995). Regardless of the fundamental attitude to and opinion on the social media, mutual respect between diverse users and good manners make the foundation of moral and appropriate behaviour (Uusiautti & Määttä, 2014a). It is necessary to think of the reasons and ways of action in these environments, and if they require specific consideration, these should be explicitly brought out so that the usage could become somehow clear and demystified. First and foremost, the possible implications for enhancing well-being and positive development should be thoroughly studied. This study highlighted the benefits of SNSs to students' social capital.

SNSs make a natural part of adolescents' lives today. Then again, SNSs are not for everyone and no one should be forced to join these communities, nor is the influence of online peer support perceived positively by everyone axiomatically (e.g. Takahashi et al., 2009). In addition, social media is not necessarily available for everyone due to technical reasons (e.g. unavailability of an internet access) or principles (e.g. some people are against SNSs) (e.g. Norton, 2012), and these issues must be realised and respected. The risks must be realised, too, and not belittled: In the social media, people act at the limits of reality and, sometimes, imagination takes over and people construct an online personality that does not correspond to themselves in the real life (Buffardi & Campbell, 2008; Panek, Nardis, & Konrath, 2013).

However, what came apparent from the findings, is that research on more efficient and educative usage of social media are needed. Further studies could also discuss the development of positive psychological capital via social media. While threats and dangers must be realised, it would also reasonable to rigorously study the possible benefits of social media, too, as the SNSs keep increasing the number of their users.
